# Nanoparticulate drug delivery systems for the treatment of neglected tropical protozoan diseases

**DOI:** 10.1590/1678-9199-JVATITD-1441-18

**Published:** 2019-02-11

**Authors:** Greta Volpedo, Lourena Costa, Nathan Ryan, Gregory Halsey, Abhay Satoskar, Steve Oghumu

**Affiliations:** 1 Ohio State University Wexner Medical Center, Department of Pathology, Columbus, OH, 43210, USA.; 2 Ohio State University, Department of Microbiology, Columbus, OH, 43210, USA.; 3 Universidade Federal de Minas Gerais, Faculdade de Medicina, Departamento de Infectologia e Medicina Tropical, Belo Horizonte, MG, Brasil.

**Keywords:** Leishmaniasis, Chagas, Trypanosomiasis, Nanoparticles, Liposomes, Nanotubes, Nanodiscs, Micelles

## Abstract

Neglected Tropical Diseases (NTDs) comprise of a group of seventeen infectious conditions endemic in many developing countries. Among these diseases are three of protozoan origin, namely leishmaniasis, Chagas disease, and African trypanosomiasis, caused by the parasites *Leishmania spp.*, *Trypanosoma cruzi*, and *Trypanosoma brucei* respectively. These diseases have their own unique challenges which are associated with the development of effective prevention and treatment methods. Collectively, these parasitic diseases cause more deaths worldwide than all other NTDs combined. Moreover, many current therapies for these diseases are limited in their efficacy, possessing harmful or potentially fatal side effects at therapeutic doses. It is therefore imperative that new treatment strategies for these parasitic diseases are developed. Nanoparticulate drug delivery systems have emerged as a promising area of research in the therapy and prevention of NTDs. These delivery systems provide novel mechanisms for targeted drug delivery within the host, maximizing therapeutic effects while minimizing systemic side effects. Currently approved drugs may also be repackaged using these delivery systems, allowing for their potential use in NTDs of protozoan origin. Current research on these novel delivery systems has provided insight into possible indications, with evidence demonstrating their improved ability to specifically target pathogens, penetrate barriers within the host, and reduce toxicity with lower dose regimens. In this review, we will examine current research on these delivery systems, focusing on applications in the treatment of leishmaniasis, Chagas disease, and African trypanosomiasis. Nanoparticulate systems present a unique therapeutic alternative through the repositioning of existing medications and directed drug delivery.

## Background

### Neglected tropical diseases 

Neglected Tropical Diseases (NTDs) represent a group of seventeen diseases caused by viruses, bacteria and parasites. NTDs are responsible for a substantial portion of the global health burden and affect more than a hundred nations, primarily developing countries and areas where communities live in poor sanitary and hygienic conditions [1] (http://www.who.int/neglected_diseases/diseases/en/ ; https://www.cdc.gov/globalhealth/ntd/index.html ). The health burden caused by NTDs is often accompanied by a financial challenge, as developing effective public health approaches to control the disease costs billions of dollars per year [2]. Furthermore, these diseases can cause long-lasting effects that can prevent infected individuals from earning a living, impacting the already precarious socio-economic situation of many communities [2-4]. Despite the efforts of the international community to control these diseases, NTDs are still a long way from being completely eradicated. Some of the issues related to this are tied to the fact that NTD endemic areas often have unstable political situations and can be subjected to military unrest [5]. For example, social instability and turmoil following the independence conflicts in many African countries between the 1980s and 1990s, led to a rapid resurgence of *Trypanosoma brucei* infections [3]. In addition to this, NTD endemic areas are often geographically restricted and therefore do not attract the attention they deserve by the global community [5]. Out of the 17 official NTDs, this review will focus on the three protozoan diseases leishmaniasis, Human African Trypanosomiasis (HAT) and Chagas disease, as these three conditions are single handedly responsible for the highest number of deaths among all NTDs [6].

## Leishmaniasis

### *a. Epidemiology*

Leishmaniasis is caused by *Leishmania* protozoan parasites and is transmitted by female Phlebotomine sandflies. Leishmaniasis affects over 10 million people in more than 90 tropical and sub-tropical countries in the new and old world [2, 7]. Human infection is mediated by about 21 species of *Leishmania* parasites and can take three different forms [8]. The most life-threatening form is visceral leishmaniasis (VL) with an estimated incidence of about 400,000 cases per year and a mortality rate of up to 95% if left untreated and up to 10% even when treated [9]. VL can be associated with an acute or chronic infection characterized by fever, anemia and swelling of the spleen and liver [7, 9, 10]. The most widespread form is cutaneous leishmaniasis (CL), with an incidence of about 2 million cases every year. Although the risks for fatality are very low with this infection, CL leads to large lesions that can result in disfiguring scars after healing [8]. Lastly, muco-cutaneous leishmaniasis (MCL) affects the mucosal tissue of mouth, nose and throat and can lead to the partial or total disintegration of these tissues [7, 8]. CL and MCL can cause disabilities and their clear manifestations are often a reason for stigma and prejudice in affected communities [2, 8].

### *b. Immunity*

An area of active research is the study of host immunology against protozoan NTDs. Understanding the immunological processes at play during parasitic infection is crucial for the development of new therapeutics and prophylactic treatments [11]. In general, protective immunity against leishmaniasis is associated with a Th1 immune response. Th1 mediated cytokines such as interleukin (IL) 12, tumor necrosis factor alpha (TNF-α) and interferon gamma (IFN-γ) have been shown to be critical for the control of this infection [8]. Macrophage activation by Th1 cytokines such as IFN-γ, elicits the production of nitric oxide (NO), important for parasite killing [8, 12]. In leishmaniasis, a Th2 immune response characterized by IL-10 production is often associated with disease susceptibility [13]. Along with Th2, B cell responses are also associated with susceptibility [14].

### *c. Prevention & Treatment*

For many years there has been considerable effort to find a candidate prophylactic vaccine for the protozoan NTDs. Some promising candidates for leishmaniasis include live attenuated vaccines [15 -18], protein fraction vaccines [15, 19], phage therapy [20, 21], DNA vaccines [15, 22 -24], chimeric vaccines [15], etc. Low market profitability, failure to confer a long lasting immunity, difference in pathogenic dynamics amongst *Leishmania* species as well as adjuvant suitability remain important challenges in the development of an effective *Leishmania* vaccine [15]. 

Treatment of these three protozoan NTDs is often challenging and there are currently only a handful of therapeutics available. Pentavalent antimonials have been the golden standard for the treatment of leishmaniasis for more than half a century. Since then, more drugs like amphotericin B (AmB), paromomycin (PM), pentamidine, miltefosine, imiquimod and azoles have been approved [7]. These compounds are sometimes also used in combination with one another to increase efficacy and reduce the side effects [7]. These drugs have been associated with hypoglycemia, nephrotoxicity, pancreatitis, cardiopathy, hypotension and hepatotoxicity [25 -27]. Increasing parasite drug resistance has also become a serious concern [26, 28 -30]. 

## Chagas disease

### *a. Epidemiology*

Chagas disease is caused by the blood protozoan parasite *Trypanosoma cruzi* and is transmitted by contact with feces of infected triatomine bugs (kissing bugs) [31 -33]. Chagas disease is prevalent in Central and South America, affecting about 8 million people and causing around 20,000 deaths each year (CDC Website) [34, 35]. It is estimated that 25% of the population of Latin America is at risk for infection [34] and that 300,000 infected people currently live in the US [36, 37]. Just like leishmaniasis, Chagas disease is mainly a threat to those living in poor sanitation and in contact with insect vectors and reservoirs [37]. In the acute phase of the disease, lasting 4 to 8 weeks, patients usually manifest mild or no symptoms [38, 39]. After this initial phase, 20 to 30% of patients progress to a chronic infection [37]. This phase is characterized by cardiac, digestive and/or neurological pathologies that can lead to systemic and pulmonary embolisms and in the most severe cases, sudden death [37, 39]. 

### *b. Immunity*

Similar to leishmaniasis, resistance to Chagas disease is mediated by a Th1-polarized immune response, which in turn activates innate immune cells [11, 16]. Along with Th1 immunity, CD8+ and Th17-mediated responses are characteristic of protection against Chagas disease [40, 41]. Differently from leishmaniasis, in Chagas disease, a balance between a pro and anti-inflammatory response seems necessary once the infection moves onto the chronic stage, the former to limit parasitic replication and spread of the infection, the latter to control and repair tissue damage [39, 42]. 

### *c. Prevention & Treatment*

There has been considerable effort for the development of a vaccine against Chagas disease. Killed and attenuated vaccines as well as cell fraction, purified protein, recombinant protein and DNA vaccines have been tested [43]. Current studies have found evidence suggesting a role for CD8+ T cells as vaccine effectors [44]. Although these strategies remain feasible, there are still some challenges to overcome. For instance, the use of protein vaccines, both purified and recombinant, are costly and time consuming. Additionally, DNA vaccines often need to be co-administered with immunostimulators to enhance their efficacy [43]. 

There are currently only two treatments available for Chagas disease: nifurtimox and benznidazole (BZN); these drugs frequently present adverse dermatological effects and can cause fever or lymphadenopathy [45]. Gastrointestinal disturbances such as anorexia, weight loss, nausea, vomiting, and abdominal discomfort are also common [45]. Associated neurotoxicity can cause irritability, insomnia, disorientation, and infrequently induces tremors [45]. More serious but less common adverse effects include paresthesia, polyneuropathy, and peripheral neuritis [45]. 

## Human African Trypanosomiasis:

### *a. Epidemiology*

Human African trypanosomiasis (HAT), better known as “sleeping sickness”, is caused by protozoan parasites of the species *Trypanosoma brucei* and is transmitted by the tsetse fly, found in sub-Saharan Africa [46, 47]. HAT can present itself in two different forms, depending on the subspecies of infective parasites. *Trypanosoma brucei gambiense* is responsible for the chronic form of the disease and accounts for 97% of reported cases in more than 20 countries [3, 46, 47]. On the other hand, *Trypanosoma brucei rhodesiense* is present in 13 countries and causes acute infections that can rapidly progress to affect the nervous system [3, 46, 47]. The first stage of HAT infection is the hemo-lymphatic stage in which the parasites replicate in the blood and lymph [3, 46, 47]. This primary phase is characterized by fever, headaches, and joint pains. Following this early stage, the parasites may cross the blood brain barrier and infect the central nervous system. This infection is referred to as the meningo-encephalic stage and is accompanied by neurological symptoms like confusion, behavioral changes, impaired coordination and sleep cycle disturbances [3, 46, 47]. When left untreated this disease can lead to coma and ultimately death by multiple organ failure [3]. 

### *b. Immunity*

Pro-inflammatory innate and adaptive responses analogous to those observed in leishmaniasis and Chagas disease, are also crucial for the control of *T. brucei* infection [19-21]. While an early Th1 response is important for resistance, the development of a late Th2 response can mediate tissue repair and is therefore beneficial in HAT patients [39, 42]. B cells also play an important role against *T. brucei*, as they release antibodies responsible for phagocytosis as well as classical pathway-mediated complement lysis [42, 48].

### *c. Prevention & Treatment*

Vaccine development to prevent *T. brucei* infection has also been accompanied by many challenges. A variable surface glycoprotein (VSG) coat continues to be the largest hurdle, preventing the development of any sort of prophylactic for HAT. Because of their clear role in pathogenesis, conserved regions in VSGs have been identified as potential antigenic vaccine targets [49], although these proteins might be more diverse than originally anticipated due to phenotypical clonal plasticity, making it difficult to identify widely “conserved” regions [49].

The current treatments for HAT are comprised of distinct and specific types of drugs for each of the two stages of *T. brucei* infection [50]. *T. brucei* acute infection can be treated by pentamidine or suramin, depending on the species, while the chronic phase can be controlled with melarsoprol or eflornithine plus nifurtimox [47]. These drugs can present serious side effects; for example, pentamidine treatment can lead to metabolism imbalance, cardiac and digestive problems while suramin can cause renal, neurological, epithelial, and bone marrow toxicity [47]. *T. brucei* treatments were developed nearly 50 years ago, are painful and can cause harmful side effects that may require hospitalization or cause death [47].

Because of the desperate need to find new prophylactic and therapeutic compounds to control these three protozoan NTDs, a considerable effort has been made to develop new potential therapeutics as well as screening for new drug targets. Due to the high cost and long timeline associated with novel drug development, it is often preferable to invest in drugs already used and approved. An approach is to change the formulation or delivery system of a drug and use it for the same disease it was originally developed for. The use of novel delivery systems can result in increased efficiency and reduced toxicity and side effects. Additionally, absorption of drugs with low solubility in water can be improved by encapsulating these compounds in lipid systems. This review will focus on lipid, polymeric and metallic/inorganic drug delivery methods for the treatment of leishmaniasis, HAT and Chagas disease. Our objective is to provide an overview of the different nanoparticulate delivery systems available for compounds that have been tested and shown to be effective for the treatment of the three protozoan NTD’s.

## Nanoparticle drug delivery systems

**Figure 1:**
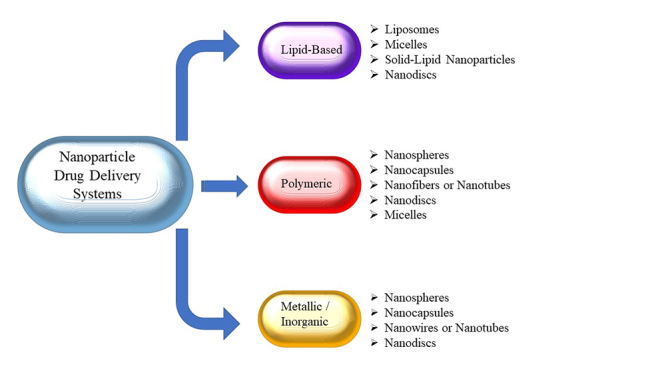
Overview of the relationship between nanoparticulate systems, divided by their structural organization.

Recently, several research groups have incorporated existing therapeutics into nanoparticulate systems. While there are no internationally agreed definitions for nanoparticles, the Publicly Available Specification (PAS71), a document developed in the United Kingdom, describes a nanoparticle as a body having one or more dimensions of the order of 100 nm or less. Nanoparticulate systems currently in use involve, but are not limited to, lipid systems and polymeric/metallic constructions (Figure 1) [51]. There are several methods of action used by these nanoparticulate systems to specifically target tissues or cells. One of these mechanisms is through direct binding to particular cell surface receptors that may be overexpressed on the desired target [52]. Nanoparticles may also diffuse to tissues through passive permeability. Increased permeability of certain tissues due to higher metabolism rates can allow for the accumulation of nanoparticles, enhancing bioavailability [52]. Here, we briefly review the use of nanoparticulate systems in the treatment of tropical parasitic conditions (Figure 1).

## Lipid systems

 Amongst the nanoparticulate systems, the lipid particles are the simplest to produce on a large scale. They usually have a biocompatible profile, as their composition presents similarity to plasma membrane lipids and cholesterol of human beings and other vertebrate animals. Lipid nanoparticles are biodegradable in the environment and have low toxicity, especially when compared to polymeric particles [53, 54].

Lipid nanoparticles are administered through different routes notably: topical, ocular, oral, pulmonary, parenteral and cerebral. The topical route presents some limitations such as the difficulty of penetration in the skin / dermis, which depends on several factors such as lipophilicity, hydration and thickness of the epidermis. Ocular administration is a good option but, due to the complexity of the eye, the bioavailability of the drug is inconsistent. The particles must be able to overcome several barriers such as the mucosa, the corneal and conjunctival epithelium, and the ocular drainage. The oral route is one of the most used, due to simplicity of administration and good acceptability. However, the first-pass (hepatic) metabolism can greatly reduce the bioavailability of the drug. The pulmonary route is newly tested and is extremely interesting because it is noninvasive and at the same time has good systemic bioavailability and presents low adverse effect. Lipid nanoparticles such as solid lipid nanoparticles (SLNs) and nanostructured lipid carrier (NLCs) can maintain high and constant levels of drugs in the blood (constant plasma levels). For the parenteral route, many studies use intravenous, intramuscular, subcutaneous and direct administration applied to specific organs. Cerebral administration has the potential to overcome the blood-brain barrier and maintain optimal levels of drug delivery in the cerebral capillaries. For this route, it is necessary that the particle be very small on the order of micrometers or less than 100 nm [53, 55].

The solubility of nanoparticles is inversely proportional to their size. For instance, solubility can be increased by reducing the diameter of a particle from micro to nanometers. Nanoparticles vary in size (< 100 nm); for instance, SLNs have submicron (< 1000 nm) particle size but usually grow during storage time. These nanoparticles are produced by double emulsion: water-oil-water or by single / single oil-water. They can also be prepared by the solvent diffusion method with ethanol and anti-solvent water. These nanoparticles are manufactured from solid or liquid solid and liquid lipids and stabilized by emulsifiers. Lipid nanoparticles can incorporate both hydrophilic and hydrophobic drugs [53, 55].

**Figure 2:**
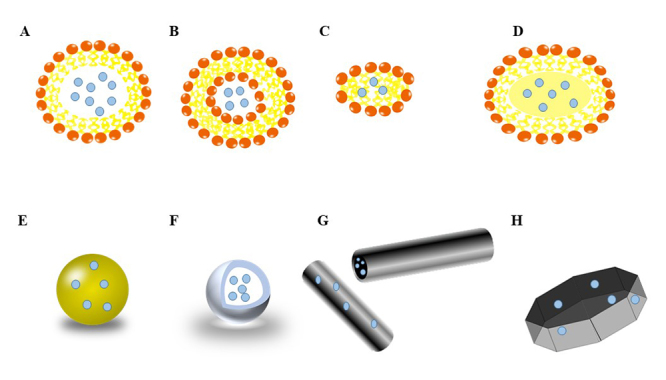
Graphical representations of the structure of various nanoparticulate systems.

Lipid systems (Figure 2 A-D) consist of vesicular structures formed by phospholipids; the most common representatives are liposomes (Figure 2B) and micelles (Figure 2A). Liposomes are small spherical vesicles divided into two major groups: the first based on structural parameters, the second on the method of preparation of the liposome [51]. Both groups are then subdivided in 8 subclasses (Table 1). Several liposomal encapsulated drugs are currently approved by the Food and Drug Administration (FDA) [56-58].

(A-D). Lipid Based Systems **(A).** Micelles **(B)**. Liposomes **(C).** Lipid Nanodiscs **(D).** Solid Lipid Nanoparticles (SLN). **(E-H).** Polymeric and Metallic / Inorganic Systems **(E.)** Nanospheres **(F.)** Nanocapsules / Polymeric Micelles **(G.)** Nanofibers and Nanotubes **(H.)** Nanodiscs.

**Table 1:** Classification of liposomal delivery systems according to molecule size and mode of preparation.

Structural Parameters	Method of Preparation
Multilamellar large vesicles (MLV) > 0.5µm	OLV made by reverse-phage evaporation (REV)
Oligolamellar vesicles (OLV) 0.1 - 1µm	MLV made by reverse-phage evaporation (MLV-REV)
Unilamellar vesicles (all sizes) (ULV)	Stable plurilamellar vesicles (SPLV)
Small unilamellar vesicles (SUV) 20 - 100nm	Frozen and thawed MLV (FATMLV)
Medium - sized unilamellar vesicles (MUV)	Vesicles prepared by extrusion (VET)
Large unilamellar vesicles (LUV) > 100nm	Vesicles prepared by French press (FPV)
Giant unilamellar vesicles (GUV) with diameters > 1µm	Vesicles prepared by fusion (FUV)
Multivesicular vesicles (MVV) >1µm	Dehydratation-rehydratation vesicles (DRV)

Micelles are generally globular aggregates however these structures may be ellipsoidal, cylindrical, or layered. Micelles may be based on lipids or polymers such as chitosan, chondroitin, or polyethylene glycol (PEG) [51]. Some micellar drug therapy formulations exist and already have FDA approval [58]. 

## Liposomes 

Changing the formulation or delivery system of a drug is referred to as repositioning and it could be useful to increase the efficacy while reducing the toxicity. Using this approach, a recent study showed that miltefosine-encapsulated liposomes are efficient in the treatment of *L. major* [59]. Another study [60] investigated the action of buparvaquone *in vitro* and determined the efficacy of liposomes charged with this drug in hamsters infected with *L. infantum* [60]. Buparvaquone liposomes (BPQ-LP) induce a Th1-type protective immune response over the Th2 response by increasing the levels of cytokines such as tumor necrosis factor and monocyte chemoattractant protein 1 (MCP-1), assisting in the elimination of parasites via production of NO. These results showed that BPQ-LP is a safe and effective treatment for murine leishmaniasis that could potentially also be used against human VL [60]. Liposomes with immunomodulatory properties can present synergistic effect with the drug they encapsulate, have beneficial effects in a variety of different diseases, and seem therefore superior to those that serve merely as delivery systems. 

Traditional liposomes (Figure 2B) have been followed by modified vesicles such as transferosomes, also called elastic vesicles. Transferosomes are made of phospholipids and contain an edge activator, which can mediate bilayer deformability. This characteristic allows transferosomes to squeeze in tight places and move through intact skin. Bavarsad, *et al.* showed that topical treatment with paromycin sulfate transferosomal formulations was more effective than the drug alone and led to smaller lesions and decreased parasitic burden in mice infected with *L. major* [61].

Another study from Morilla, *et al*. [62] designed multilamellar liposomes (MLV) composed of hydrogenated soybean phosphatidylcholine, distearoyl-phosphatidylglycerol, and cholesterol, loaded with BZN, a currently approved treatment for Chagas disease. This formulation was more rapidly eliminated from the circulation compared to the free drug. Additionally, due to decreased interactions of this new formulation with plasma proteins and other blood components, the toxic side effects were also reduced. This data shows that MLV BZN is a vehicle potentially capable of improving the treatment with BZN against *T. cruzi* infection [62]. Another preliminary study [63] showed the superior efficacy of a liposomal preparation of diminazene aceturate, compared to the free compound, *in vitro* against *Trypanosoma evansi*, a trypanosome causing equine illness similar to *T. brucei* in humans. However, *in vivo* testing was inconclusive, and further studies are required to find a more effective liposomal preparation with greater *in vivo* efficacy [63]. It has been shown that lipid nanoparticle formulations of diminazine lead to increased trypanocidal activity against *T. b. brucei* as well as an increased stability *in vitro*, due to protection from oxidation [64].

## Micelles

Micelles (Figure 2A) have been used in a study evaluating the *in vitro* and *in vivo* anti-leishmanial activity of 8-hydroxyquinoline in a micelle system against *L. infantum*. This micelle delivery system led to significant reduction in parasitic burdens and elicited a Th1 immune response, indicated by high levels of IFN-γ, IL-12, granulocyte-macrophage colony-stimulating factor, nitric oxide and anti-leishmanial IgG1 isotype antibodies [65]. Furthermore, the interaction of 5-nitro-2-furfurilylidene benzhydrazide, a potential anti-trypanosomal compound with micellar solutions, was evaluated by Rangel-Yagui, *et al* [66]. In this study, the compound was shown to be five times more effective than BZN, the drug of choice for Chagas disease [66]. In a comparative *in vivo* study of a micellar diminazine formulation against *T. evansi*, micellar preparations had a higher drug accumulation rate within erythrocytes through greater cell membrane interaction. This resulted not only in greater efficacy, but also allowed for a decreased dose requirement *in vivo*, indicating great promise for similar preparations made against *T. brucei* in the human host [67].

## Solid lipid nanoparticles (SLN)

SLN (Figure 2D) were first developed in 1996 and can be classified into three types of structures: SLN, nanostructured lipid carrier (NLC), and lipid-conjugates. These particles are solid at room and body temperature, and compromised of lipids such as glyceryl monostearate and stearic acid [51]. Because SLN contain lipids, they retain a biocompatible and biodegradable profile, but because they are solid and rigid, they allow good protection of the drug incorporated, even in inhospitable environments with great variation of pH, humidity and temperature. SLN have low drug loading efficiency because of the restricted allosteric space. SLN are typically produced using a high pressure homogenization technique. During the storage process, crystallization may occur, which results in drug expulsion from the SLN [68]. Nanostructured lipid carriers (NLCs) are similar to SLN but have a greater ability to incorporate drugs, do not usually crystallize during storage processes and have liquid lipids inside them instead of solid [69]. These particles are ideal for delivering hydrophobic drugs as they are composed entirely of lipids.

A recent study [70] associated SLN with PM, an anti-leishmanial drug, to improve its effectiveness. PM-SLN were evaluated *in vivo* against infection of *L. major* in BALB/c mice. PM-loaded SLN have greater apparent safety compared to the free drug and are more efficient as they increased penetration of the drug into macrophages in addition to enhancing the immune response [70]. In particular, PM-loaded SLN were more efficient than free PM for the treatment of *L. tropica* and the augmentation of a protective immune response in mice [71]. Searching for new therapeutic drugs against *T. cruzi*, Carneiro, *et al.*[72] evaluated *in vitro* and *in vivo* efficacy of 5-hydroxy-3-methyl-5-phenyl pyrazoline-1 (S-benzyl dithiocarbazate) as a free substance and inside an SLN system. The nanoparticles were effective in reducing the parasitic loads in infected animals and also reduced the inflammatory process in the liver, spleen and heart, and still promoted a survival of 100% of the infected mice [72]. SLN have also shown promising results for the treatment of *Trypanosoma spp.* infections. In a study comparing *in vivo* effects of a SLN α-bisabolol preparation using standard diminazine aceturate treatment as well as combination therapy in a *T. evansi* model, it was found that combination therapy with SLN preparations greatly enhanced curative effects compared with either drug taken alone [73]. Combination therapy such as eflornithine-nifurtimox therapy is currently widely used for the treatment of *T. brucei* infections and so this potential use of SLN to amplify the curative properties of different drugs is of particular interest [74]. Furthermore, nanostructured lipid carrier (NLC) systems can improve efficacy of poorly water-soluble drugs against protozoan diseases. For instance, NLC carrying cedrol have shown improved antiparasitic activity against *L. donovani in vitro* and *in vivo* compared to treatment with the free drug [75].

## Nanodiscs

Lipid nanodiscs (Figure 2C) are composed of a phospholipid bilayer surrounded by the amphiphilic motifs of apolipoproteins [76]. The efficiency of nanodiscs harboring the toxic and poorly soluble anti-leishmanial agent AmB was assessed in *L. major*-infected BALB/c mice with increased efficacy observed. These results identify nanodiscs as potentially useful delivery systems for the treatment of intra-histiocyte pathogens [76]. Nanodiscs possess similar characteristics to micelles but are smaller and simpler in structure, possibly allowing for easier customization of lipid composition and apolipoproteins as well as more effective cellular internalization.

## Polymeric systems

Polymeric systems (Figure 2E-H) include polymeric nanoparticles such as nanocapsules (Figure 2F) and nanospheres (Figure 2E): which are solid particles in the colloidal state, formed by polymeric materials. The method of preparation may favor the formation of one or the other pharmaceutical forms. Nanospheres are composed of a spherical polymeric matrix, whereas nanocapsules have a polymeric wall and an oleaginous-oily nucleus [77, 78]. Some polymeric systems have already been approved by the FDA to be used in the treatment of a wide array of diseases [58, 73, 74]. Polymeric particulates are of great interest due to diverse applicability and their ability to act as bio-conductors [79].

## Nanocapsules and Polymeric Micelles

Asthana, *et al*. show that mannose grafted chitosan nanocapsules (MnosCNc) containing AmB, have high specificity for macrophages, the preferred host cell for *Leishmania*, and present antiparasitic activity against *L. donovani in vitro* and *in vivo* [80]. Interestingly, lipid-core nanocapsules have also been used to improve the efficacy of Quercetin against *L. amazonensis* [81]. In an animal model of HAT, quinapyramine sulfate encapsulated within nanoparticles consisting of chitosan, tripolyphosphate, and mannitol or sodium alginate was shown to have stronger *in vivo* efficacy against *T. evansi* than the drug alone, even when lower doses were administered to animals [82-84]. Further, this drug is intended for animal use only, as its side effects are so severe, and this nanocapsule formulation reduced the side effects in animals to a negligible amount. Nevertheless, this reduction in negative side effects in the host demonstrates the need for further study in this area, as there is the possibility that efficient trypanocidal drugs previously overlooked due to severe side effects may be administered in nanocapsule form to mitigate any ill effects. Using a chitosan nanoparticle coated with a nanobody specific for the cell surface of *T. brucei*, Garcio-Salcedo *e. al.* was able to administer pentamidine in a murine *T. brucei* model at an effective dose 100-fold lower than the effective dose for the drug alone. Direct targeting allowed for the uptake of the drug through endocytosis, resulting in a drug effective against drug resistant strains [85]. Nanobody formulations such as this may have applications against *Leishmania* as well, as macrophage specific nanobodies may be utilized to administer anti-leishmanial drugs specifically into the host’s macrophages, increasing access to amastigotes. Several studies have characterized various encapsulated formulations of BZN, the current first-line drug for the treatment of Chagas disease. Novel nanostructure BZN carrier efficacy was examined in fibroblasts *in vitro* as well as *in vivo* murine models resulting in reduced mammalian cell cytotoxicity, decreased parasite viability and increased survival rates of infected mice [86-89]. Similarly, lychnopholide encapsulated in polymeric nanocapsules was effective in curing chronic phase infection *via* oral administration and was just as effective as standard BZN treatment during acute phase *via* intravenous administration in murine models [90, 91]. 

Nanoencapsulated natural remedies, including tea tree oil, curcumin, and *Achyrocline satureioides* oil, have all shown greater efficacy against different trypanosome species than non-carrier-based formulations [92-94]. Nanoencapsulated compounds showed greater trypanostatic or trypanocidal effect against *T. evansi*, with a reduction in side effects and effective dose. Furthermore, nano-encapsulated tea tree oil was shown to improve the *in vivo* efficacy of diminazine aceturate from just 33% to 100%, demonstrating the need for further research in drug combination studies involving nano-encapsulated drug formulations.

Polymeric micelles are another type of nanomaterial that has attracted considerable attention as a potential drug carrier. Micelles form spontaneously in aqueous solutions and are composed of amphipathic copolymers. The use of polymeric micelles such as Poloxamer P407 (Pluronic® F127) (Amp/M) and the Ambisome® formulation (Lip-Amp) have been evaluated as delivery systems for leishmaniasis treatments. It was demonstrated that Amp/M or Lip-Amp treated mice had lower parasitic burden along with a more prominent Th1 immune response compared to the control mice treated with the free drugs; additionally, micelle formulation-treated mice did not develop hepatic or renal damage [95].

While polymeric nanocapsules and micelles have similar initial burst drug release kinetics to lipid-based micelles, there is a time delay before drug release as the external polymeric membrane must be degraded. Tuning of polymeric membrane properties such as attached functional groups, molecular weight, and zeta potential allows more customization of drug release than offered by traditional lipid-based micelles [96].

## Nanospheres

Nanospheres constructed using chitosan and chondroitin containing AmB (NQC-AmB) have been studied and tested in animals infected with *L. amazonensis,* the causative species of CL in the Americas. AmB loaded nanosphere treatment reduced parasitic loads, lesion size, and drug toxicity compared to treatment with free drug alone, indicating that the NQC-AmB system was effective in directed delivery [97]. Interestingly, hemoglobin-guided NQC-AmB has shown reduced LD50 and toxicity in macrophages infected with *L. donovani* compared to those treated with AmB alone [98]. Furthermore, PLGA-PEG encapsulated AmB nanospheres have been shown to be more effective and less toxic than free AmB for the treatment of *L. donovani* infection [99]. Similarly, PLGA-encapsulated desoxycholate AmB was more effective than the free drug for the treatment of mice infected with cutaneous leishmaniasis [100]. Another study showed that artemisin-loaded nanospheres significantly reduced parasitic burdens and hepato-splenomegaly in mice infected with *L. donovani* compared to the group treated with the free drug. These effects were associated with an augmented Th1 response, suggesting that this formulation also possesses immunomodulatory properties [101]. 

Chitosan nanoparticles (CS NPs) synthesized together with the precursor of nitric oxide in mercaptosuccinic acid (MSA) have been studied in a Chagas disease model. Treatment of peritoneal macrophages with these nanoparticles decreased the number of *T. cruzi* infected cells and the average number of intracellular replicative amastigotes per infected cell. These results suggest that the S-nitroso-MSA-CS NPs are promising nanocarriers for the treatment of Chagas disease and can deliver nitric oxide intracellularly [102]. Ursolic acid loaded poly-ε-caprolactone nanoparticles have also been evaluated *in vivo* showing similar efficacy to BZN standard formulations [103]. Polymeric antibody delivery systems have also been developed to target *T. b. gambiense*. PEG covalently attached to PLGA and loaded with pentamidine alongside an antibody fragment specifically targeting the *T. brucei* cell wall has shown positive results both *in vitro* and *in vivo* with 100% cure rate of *T. b. gambiense* infected mice. This outstanding result is even more remarkable as it was obtained using only a tenth of the normal dose of pentamidine. Additionally, a 100-fold decrease in drug concentration still resulted in a 60% cure rate with this nanosphere delivery system [104]. Polymeric nanospheres contain an even distribution of a given drug throughout the solid polymer matrix and drug release is timed in coordination with matrix degradation and diffusion of the drug. This characteristic makes these particles more suitable for sustained drug release applications, providing a useful application during chronic infection by these parasites or in situations where resurgence of the parasite burden may occur [96].

## Nanofibers

Recent advances in materials science and biochemistry have allowed for the construction of high aspect ratio nanofibers (Figure 2G) using self-assembling peptide amphiphiles. These nanofibers may be incorporated with a targeting motif and have demonstrated ability to adhere to *in vivo* targets with high specificity. Functionalization of these nanofibers by S-nitrosylation reaction of the cysteine residues of the PAs allowed for targeted and sustained delivery of nitric oxide to a specific site determined by the binding target of the attached targeting-sequence [105].

## Metallic / inorganic systems

Metallic/inorganic systems (Figure 2E-H) have been recently developed and include inorganic nanoparticles, like carbon nanotubes, as well as metallic (metal and metal oxide) nanoparticles composed of different metals such as gold, silver magnesium, titanium, and zinc. Drugs are adsorbed or covalently bound to the surface of these particles which exist as a colloidal suspension in liquid. Drug release from metallic nanoparticles is dictated by the strength of these bonding forces between the desired drug and the surface of the nanoparticle; however, coating of the particle surface with polymers can alter these characteristics. An advantage of metallic systems over other nanoparticle systems is that it is easier to locate cells containing nanoparticles *in vivo* post-administration using imaging techniques [106, 107].

## Nanospheres

Recent studies have validated the antimicrobial potential of various metals and metal oxides. Jebali, *et al*. tested different metallic nanoparticles in a CL model caused by *L. major,* aiming to understand the anti-microbial effects of nanoparticles in the presence or absence of ultra violet (UV)/pIR light utilizing gold (Au NPs), silver (Ag NPs), magnesium (MgO NPs), titanium (TiO2 NPs) and zinc oxide (ZnO NPs) particles. Their findings suggest great potential for these metallic particles in antimicrobial use, which is enhanced by exposure to either UV or IR light [108]. Ag NPs have been identified as a potential therapeutic option as they cause damage to the structural integrity of intracellular *L. amazoniensis* amastigotes [109]. ZnO NPs have also showed potential antiparasitic activity against *L. tropica* in vitro, with an IC_50_ up to 3.76 against promastigotes [110]. A different study [111] investigating the effect of quercetin conjugated AuNP (QAuNP), found that QAuNP efficiently targeted *Leishmania* amastigotes with a high selectivity index, an indispensable requirement to approve the use of a drug [111]. Potential anti-trypanosomal drugs have also been identified in the bioactive pigment molecules prodigiosin and violacein. These two drugs both showed efficacy against *T. b. gambiense in vitro* and represent a possible new therapeutic against HAT. When these compounds were prepared with metal nanoparticles, IC50 values significantly decreased. In the same study, similar results were seen when this preparation was tested against another pathogenic parasite, *Plasmodium falciparum,* the causative agent of malaria [112]. 

Gold and silver nanoparticles have been shown to have high specificity and binding affinity towards arginine kinase of *T. brucei* [113]. Further, Kato *et al*. have demonstrated the trypanostatic effect metal nanoparticles have against different species of trypanosome, particularly *T. brucei*, with high selective activity [114]. Taken together, these observations highlight the potential of metal nanoparticles, particularly gold and silver nanoparticles, against *Trypanosoma spp*. With high specificity for a kinase present only in *Trypanosoma spp*., these nanoparticles may be incorporated into other drug formulations, resulting in formulations specifically targeting arginine kinase, and the trypanosomes, allowing for reduced IC50 values and a mitigation of possible side effects for the host.

## Nanotubes

Nanotubes (Figure 2G) are hollow structures which can be formed by inorganic materials such as carbon, titanium, as well as metallic materials like gold and silver. A study examining the anti-leishmanial efficacy of a novel formulation of AmB associated with carbon nanotubes (CNTs), found enhanced targeted killing of *Leishmania donovani in vivo* and *in vitro* compared with free AmB [115]. Furthermore, a group from India that has developed a new anti-leishmanial formulation of betulin (BET) associated with CNTs, reported that CNTs with BET showed better cytotoxicity and IC 50% compared to the control group, further supporting the possibility of nanotubes as an effective drug delivery system [116]. The efficiency of CNTs as a delivery system was also demonstrated for the standard treatment of VL, AmB[117]. Using a hamster VL model, oral AmB administered with nanotubes exhibited 99% inhibition of parasite growth. CNTs have not been approved for use in humans and testing is at the preclinical stage [117]. Another study tested the potential of polysaccharide (mannose) associated with multi-walled CNTs plus AmB and proved that CNTs can deliver a drug to specific organs where there are elevated numbers of macrophages. These nanotubes provide a more direct and safer way to deliver the drug to the target tissue [118]. CNTs have also been used alongside a protein specific RNA aptamer for use in an aptasensor, which is able to detect proteins at the attomolar (10^-18^ mol) level [119]. This new technology applied to HAT would allow for early detection of the disease, enabling treatment prior to progression to the fatal stage II disease of the central nervous system, and allowing for earlier, more successful treatments of the infection. Once in the central nervous system of the host, treatment becomes far more difficult, as formulations of drugs capable of crossing the blood brain barrier and targeting trypanosomes are extremely limited. However, it has been shown that certain functionalized carbon nanotube formulations are able to increase the ability of a compound to cross the blood brain barrier, which could be used to treat late stage infections of HAT [120].

## Conclusion

Of the 17 official NTDs, this review focuses on the only three protozoan diseases namely, leishmaniasis, Chagas disease and HAT, and these three conditions alone are responsible for the highest death toll amongst all the NTDs [6]. Despite the efforts of the scientific community, there are very few preventive measures to control these diseases and no vaccines against these parasitic infections have been approved for human use [46, 121, 122]. Failure to confer a long-lasting immunity [15, 43, 49], as well as high cost and extended time lines [43] are major challenges against the development of an effective vaccine. Furthermore, the therapeutics available for the treatment of these three NTDs often present toxic side effects [7, 25 -27, 45, 47] and some of these drugs have been associated with increasing parasitic resistance [26, 28 -30]. Because of the shortcomings of these preventive and therapeutic measures, it is imperative to find new approaches to control these infections. 

An efficient approach is to administer an already existing drug using different and novel delivery systems to limit the toxic side effects and enhance the bio-availability and efficacy of the compound. In this review we highlighted different nanoparticulate systems used experimentally for the treatment of the three protozoan NTDs. Nanoparticulate drug delivery systems of lipid, polymeric or metallic composition have emerged as a promising area of research, as scientific evidence shows that they can improve the ability to specifically target pathogens, penetrate barriers within the host allowing a drug to access areas of pathogen residence, and reduce toxicity by lowering dose amount and frequency of administration [120, 123, 124]. In general, nanoparticulate systems can possess multiple synergistic functions while maintaining high specificity and selectivity. To improve bio-availability and efficacy, as well as to treat multiple diseases with the same formulations, scientists have been testing co-delivery systems, using the same delivery system to encapsulate multiple drugs with synergistic properties [125 -127]. Synergy is already exploited in the treatment of HAT through the combined use of nifurtimox and eflornithine, and as such these multi-drug encapsulations may be particularly effective against this parasite, though further studies are required. These delivery systems also have the potential to directly target the immune system, as well as the parasites themselves. For example, nitric oxide acts as an endogenous signaling molecule influencing host immune responses to protozoan parasites in a positive manner and promoting subsequent reduction of parasitic burden. It has been shown that systemically administered nanoparticles possess the capability to release nitric oxide at targeted sites within the body or intracellularly within macrophages [102, 105]. Given the beneficial effect of nitric oxide on protozoan disease progression, nitric oxide releasing functionalized nanoparticles present possible alternatives to traditional toxic front-line chemotherapeutic options. Furthermore, this review focused mainly on treatment strategies, but several studies suggest that lipid, polymeric and metallic nanoparticulate systems could also be used for the delivery of vaccines against the three protozoan NTDs [128, 129]. Interestingly, lipid systems have also shown promise as vaccine adjuvants, by enhancing the immune response to vaccines [130].

 Despite having many positive qualities for parasite treatment, nanoparticles must overcome some challenges to be effectively and widely employed in clinical medicine. Limitations are associated with preparation methods, elimination pathways as well as the lack of knowledge on mechanisms of action [131, 132], each of which poses as an area of interest, requiring further study by the scientific community. Nevertheless, repositioning *via* nanoparticulate systems appears to be a promising approach to reduce the risks and downfalls associated with the currently available therapeutic compounds.

## Abbreviations

**AmB**: Amphotericin B; **BZN**: Benznidazole; **BET**: Betulin; **BPQ-LP**: Buparvaquone liposome; **CNTs**: Carbon nanotubes; **CS NPs**: Chitosan Nanoparticles; **CL**: Cutaneous Leishmaniasis; **FDA**: Food and Drug Administration; **AuNP**: Gold nanoparticle; **HAT**: Human African Trypanosomiasis; **IFN-γ**: Interferon gamma; **Interleukin**: IL; **L.**: *Leishmania*; **MgO NPs**: Magnesium Nanoparticles; **MCP-1**: monocyte chemoattractant protein; **MLV**: Multilamellar vacuoles; **NQC-AmB**: Nanoparticle Chitosan, Chondroitin, Amphotericin; **NTD**: Neglected Tropical Disease; **NO**: Nitric Oxide; **PEG**: Polyethylene glycol; **QAuNP**: Quercetin-gold nanoparticle; **Ag NPs**: Silver Nanoparticles; **SLN**: Solid lipid nanoparticles; **TiO2 NPs**: Titanium Nanoparticles; **T.**: *Trypanosoma*; **UV**: Ultra violet; **VSG**: Variable surface glycoprotein; **VL**: Visceral leishmaniasis; **ZnO NPs**: Zinc oxide Nanoparticles.
